# A prospective study of peri-diagnostic and surgical wait times for patients with presumptive colorectal, lung, or prostate cancer

**DOI:** 10.1038/sj.bjc.6604819

**Published:** 2008-12-16

**Authors:** E Grunfeld, J M Watters, R Urquhart, K O'Rourke, J Jaffey, D E Maziak, C Morash, D Patel, W K Evans

**Affiliations:** 1 Cancer Outcomes Research Program, Cancer Care Nova Scotia and Department of Medicine, Dalhousie University, Halifax, Nova Scotia, Canada; 2 Department of Epidemiology and Community Medicine, University of Ottawa, Ottawa, Ontario, Canada; 3 Cancer Therapeutics Program, Ottawa Health Research Institute, The Ottawa Hospital, Ottawa, Ontario, Canada; 4 Department of Medicine, University of Ottawa, Ottawa, Ontario, Canada; 5 Clinical Epidemiology Program, Ottawa Health Research Institute, The Ottawa Hospital, Ottawa, Ontario, Canada; 6 Department of Surgery, University of Ottawa, Ottawa, Ontario, Canada; 7 Juravinski Cancer Centre at Hamilton Health Sciences, McMaster University, Hamilton, Ontario, Canada

**Keywords:** lung cancer, colorectal cancer, prostate cancer, health services, diagnosis, surgery

## Abstract

The objective of this study was to prospectively measure peri-diagnostic and surgical time intervals for patients with suspected colorectal, lung, or prostate cancer. Prospective eligible patients were referred to a regional hospital in Ottawa, Canada between February 2004 and February 2005 for diagnostic assessment of presumptive colorectal, lung, or prostate cancer. Chart abstractions were used to measure nine time intervals; the primary interval was the date of referral for diagnostic assessment to the date the patient was informed of the diagnosis. Health-related quality-of-life (HRQL) was assessed 5 days following the patient being informed of their diagnosis. The median (IQR) time for the primary interval was 71 (30–110), 37 (29–49), and 81 (56–100) days for colorectal, lung, and prostate patients, respectively (Kruskal–Wallis *P*=0.0001). This interval was significantly less for colorectal patients diagnosed with cancer than for those without cancer (median difference=59.0 days; Wilcoxon *P*=0.003). No differences in HRQL existed for patients with cancer and those without. Colorectal and prostate patients wait longer between referral for suspected cancer and being informed of their diagnosis than current recommendations. The shorter diagnostic intervals for colorectal patients with cancer suggest clinicians have an effective process for triaging patients referred for diagnostic assessment.

Patients with suspected cancer experience many waits as they progress through their diagnostic assessment and treatment. These wait times are a lightning rod in discussions about public-funded health care systems (Lewis *et al*, 2000; Sanmartin *et al*, 2000; Oliver, 2001; Thomas and Burnet, 2001; Sibbald, 2005). National-level organisations have affirmed their commitment to establishing evidence-based benchmarks for medically acceptable wait times (Canadian Society for Surgical Oncology, n.d.; Department of Health, 2000b; Health Canada, 2004; Wait Time Alliance, 2005). The majority of Canadian-based recommendations consider only a small portion of a cancer patient's experience, such as wait times for radiation therapy (Alberta Waitlist Registry, n.d.; Quebec Ministry of Health and Social Services, n.d.; Wait Time Alliance, 2005; Cancer Care Ontario, 2006) and systemic therapy (Cancer Care Ontario, 2006). United Kingdom recommendations involve primary treatment (Department of Health, 2000b) as well as the 2-week wait rule for referral of urgent suspected cancer cases (Department of Health, 2000a).

Although treatment time intervals are important, for patients the experience is one of a series of waits starting with the peri-diagnostic period (Wait Time Alliance, 2005). Moreover, the peri-diagnostic period may critically impact subsequent outcomes (Richards *et al*, 1999). Research from the EUROCARE project (Gatta *et al*, 2000; Berrino *et al*, 2007) suggests that the lower 5-year survival ratios for breast and colorectal cancer in the United Kingdom compared to Northern and Central European countries might be attributed to higher disease stage at diagnosis. These findings point to the need for timely referral processes and early diagnoses.

Most studies of wait times use administrative data (Mayo *et al*, 2001; Johnston *et al*, 2004; Rayson *et al*, 2004; Simunovic *et al*, 2005; Liberman *et al*, 2006), yet the ability to document many of the peri-diagnostic time intervals from routinely collected data is limited. Moreover, these studies usually study patients with a cancer diagnosis (Mayo *et al*, 2001; Johnston *et al*, 2004; Rayson *et al*, 2004; Liberman *et al*, 2006) and do not report wait times for those who are found not to have cancer. Wait times during the peri-diagnostic period are a time of considerable psychological stress for all patients, even those who turn out not to have cancer (Benedict *et al*, 1994; Gray, 1997). The primary objective of this study was to measure prospectively peri-diagnostic and surgical time intervals for patients with suspected colorectal, lung, or prostate cancer. The secondary objective was to assess health-related quality of life (HRQL) 1 week following cancer being confirmed or ruled out.

## Materials and methods

We conducted a study of patients referred to The Ottawa Hospital (TOH) in Ottawa, Ontario from February 2004 to February 2005 for diagnostic assessment of presumptive colorectal, lung, or prostate cancer. TOH is a university-affiliated regional hospital serving Eastern Ontario and offers the full range of health care services from primary care to tertiary cancer care. In 2004, it had 951 beds and served as the referral centre for a population of 1.12 million people and a geographic area of approximately 17 600 km^2^. The only cancer centre in the region is at TOH. In addition to referral for presumptive cancer, patients were eligible for the study if they were 18 years of age or older, provided written informed consent, were able to complete the baseline questionnaire in English or French, had no previous diagnosis of cancer in the past 5 years (except *in situ* cervix or non-melanoma skin), and had not been receiving ongoing care/surveillance from a cancer centre or tertiary care physician. The study was approved by TOH Research Ethics Board.

Potentially eligible patients were identified from clinic lists of all participating consultants (21 of 24, 87.5% of potential consultants). Consultants included gastroenterologists and general surgeons for presumptive colorectal cancer; respirologists and thoracic surgeons for presumptive lung cancer; and urologists for presumptive prostate cancer. All prospective patients were approached to participate in the study, either in person on the day of their first diagnostic consultation or by telephone within 24 h. Upon providing written informed consent, patients were enrolled in the study and followed until their first oncology consultation or 3 months, whichever came first. Following enrolment, patients completed a baseline questionnaire to provide identifying and demographic information.

**Figure 1:**
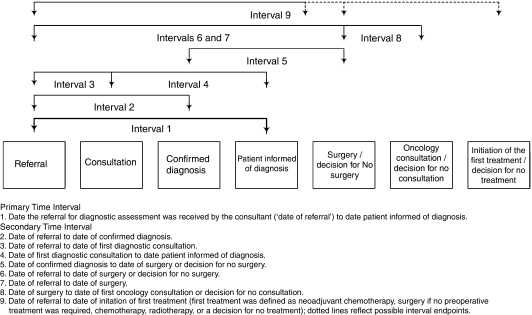
Peri-diagnostic and treatment time intervals.

Chart abstraction and patient-completed questionnaires were used to collect the data. Two experienced clinical research associates, skilled in chart abstraction, conducted the chart review. The primary time interval, calculated in days, was (1) the date the referral for diagnostic assessment was received by the consultant (based on the date of fax, letter, or telephone call received in the consultant's office, referred to hereafter as ‘date of referral’) to the date the patient was informed of the diagnosis. Secondary time intervals, calculated in days, were (2) date of referral to date of confirmed diagnosis (date of the pathology or radiology report, or date of colonoscopy for colorectal patients informed of malignancy or non-malignancy immediately after colonoscopy); (3) date of referral to date of first diagnostic consultation (or date of first relevant investigation initiated by consultant, whichever came first; relevant investigations included biopsy, bronchoscopy, chest X-ray, colonoscopy, sigmoidoscopy, CT scan, MRI, PSA, pulmonary function test, transrectal ultrasound, and other); (4) date of first diagnostic consultation to date patient informed of diagnosis; (5) date of confirmed diagnosis to date of surgery or decision for no surgery (date of decision for no surgery based on consultant notes); (6) date of referral to date of surgery or decision for no surgery; (7) date of referral to date of surgery; (8) date of surgery to date of first oncology consultation or decision for no consultation (date of decision for no consultation based on consultant notes); and (9) date of referral to date of initiation of first treatment (first treatment was defined as neoadjuvant chemotherapy, surgery if no preoperative treatment was required, chemotherapy, radiotherapy, or a decision for no treatment). See Figure 1 for a schematic of the time intervals. Patient comorbidity at the time of diagnostic assessment was calculated using the Charlson comorbidity index (Charlson *et al*, 1987).

Patients completed study questionnaires 5 days after the date they were informed of their diagnosis (either confirming or ruling out cancer), consisting of the following instruments: European Organization of Research and Treatment of Cancer quality of life core questionnaire (EORTC QLQ-C30) HRQL (30 items; range: 0–100; higher scores reflect better quality of life; Aaronson, 1995); the Hospital Anxiety and Depression Scale (HADS) to measure anxiety and depression (14 items; range: 0–21; higher scores reflect higher levels of anxiety or depression) (Zigmond and Snaith, 1983); and the SF-36 to measure physical and mental functioning (36 items; range: 0–100; higher scores reflect better functioning; Brazier *et al*, 1992). The questionnaires were mailed to patients with instructions to complete and return immediately. Received questionnaires were reviewed for completeness and patients were seen in clinic or contacted by telephone to complete missing items. If the questionnaires were not received within 3 working days, patients were seen in clinic or contacted by telephone and the questionnaire was completed at that time.

Descriptive statistics and associated analyses were performed in SAS for Windows version 9.1. Descriptive data are presented both as means and standard deviations and medians and interquartile ranges to facilitate a better description of the wait times and to enable the data to be used in future economic and other analyses. Simple logistic regression was used to compute odds ratios and 95% confidence intervals by suspected disease site for diagnosis of cancer and the attributes comorbidity, sex, and referring physician (family physician or specialist (gastroenterologists, respirologists, urologists, and surgeons)). Analyses of variance (ANOVA) were used to compare differences between disease sites for the primary time interval; *t*-tests were used to compare differences between patients with and without cancer for this same interval. Concerns about the skewness or other non-normality of continuous outcomes were addressed by sensitivity analysis using non-parametric procedures, in particular the Kruskal–Wallis rank sum test as a check on ANOVAs and the Wilcoxon rank sum test as a check on *t*-tests. The data were not log-transformed due to the objective of measuring and describing time intervals, and hence the need to maximise interpretability. For the primary time interval, parameter estimates were also computed via a multivariable linear regression model, with age, sex, comorbidity, and referring physician as the covariables. Although the data are descriptive, we followed the convention of noting statistical significance when the two-sided *P* value was less than 0.05. For comparative purposes, data are primarily reported as medians and interquartile ranges. However, as noted above, ANOVAs and *t*-tests using means and variances were used to compare differences for the primary time interval. Accordingly, *P* values should be interpreted with caution. For the questionnaire data, *t*-tests were used to compare differences between patients with and without cancer, whereas ANOVAs were used to compare differences between disease sites. A two-sided *P* value <0.05 was used to determine statistical significance.

## Results

During the study period, 461 patients met the eligibility criteria and 369 agreed to participate. Of these patients, 350 patients completed the questionnaires and represent the study population (76% participation rate). Patient characteristics are presented in Table 1. Lung patients were more likely to have comorbidities (lung *vs* colorectal: OR=2.63, 95% CI=1.52–4.55; lung *vs* prostate: OR=2.17, 95% CI=1.23–3.82) and were more likely to have been referred by someone other than a family physician (lung *vs* colorectal: OR=2.49, 95% CI=1.27–4.88; lung *vs* prostate: OR=2.86, 95% CI=1.39–5.92) than either colorectal or prostate patients. The two most common reasons for referral for each type of cancer were: change in bowel habit plus age >50 years (*n*=53) and rectal bleeding with a change in bowel habit (*n*=34) for suspected colorectal cancer; suspicious lung nodule(s) or lesion or mass on diagnostic imaging (*n*=98) and smoker plus suspicious symptoms (*n*=47) for suspected lung cancer; and increased PSA (*n*=102) and abnormal digital rectal exam (*n*=18) for suspected prostate cancer.

Lung patients were more likely to be diagnosed with cancer (80.2%) than either colorectal (6.8%) or prostate patients (35.3%; colorectal *vs* lung: 95% CI=0.008–0.04; lung *vs* prostate: 95% CI=3.93–13.60). Descriptive statistics for all time intervals are presented in Table 2. The primary outcome of this study was the time interval from the date of referral to the date the patient was informed of the diagnosis. This interval varied significantly between cancer sites: lung patients had significantly shorter time intervals (median 37 (IQR 29–49) days) than colorectal (median 71 (IQR 30–110) days) or prostate patients (median 81 (IQR 56–100) days, Kruskal–Wallis *P*=0.0001). On multivariable linear regression analysis, the level of comorbidity for lung patients was the only factor associated with differences in the interval from referral to patient informed of diagnosis (estimated coefficient=14.31; *P*=0.04). Age, sex, and referring physician were not associated with differences in this time interval.

Colorectal patients with cancer had significantly shorter time intervals between referral and being informed of diagnosis than colorectal patients without cancer (mean 33.8 (s.d. 48.9) days and mean 84.8 (s.d. 67.7) days, respectively, Wilcoxon *P*=0.003; see Table 3). There was no difference in this time interval for patients with and without suspected lung or prostate cancer.

Table 4 presents the questionnaire scores 1 week after diagnosis for patients with and without cancer. No differences in health status or HRQL existed between those patients diagnosed with cancer and those found not to have cancer.

## Discussion

Most studies of cancer diagnostic time intervals are retrospective in design, using administrative data of patients with cancer. This study is unique in prospectively measuring intervals for patients with presumptive cancer, several of which cannot be captured from routinely collected administrative data. Although it is possible to derive many time intervals from administrative data and such data permit researchers to conduct population-based studies, the gold standard is prospectively collected data from patient records. That is, the outcome of interest (eg, time intervals) is unknown at the outset of the study and measured over time, whereas the patient chart provides data that are not attainable from administrative data. The value of our approach, from a patient-centred perspective, is best illustrated by the two different definitions of the interval ‘from referral to diagnosis’: the ‘date of diagnosis’ in the first definition is the date the patient is informed, and in the second definition it is the date of the confirmatory report from pathology, radiology, or procedure (eg, colonoscopy). Considering the median for patients with cancer as an example, the difference between the first and second definitions was 0 days for colorectal cancer, 7 days for lung cancer, and 19 days for prostate cancer. Therefore, for the patient, the wait for their diagnosis can be 2.5 weeks longer than the confirmatory date, but it is only the latter date that is available from administrative data. Although there are very good reasons to use the second ‘confirmatory’ definition, including its practicality when using routinely collected data, the first definition highlights the fact that the patient may experience significantly longer waits than the administrative data reveal.

In Canada, research on wait times often comprises the surgical and treatment periods (Johnston *et al*, 2004; Simunovic *et al*, 2005), whereas most reports of diagnostic time intervals for patients with presumptive cancer involve breast cancer (Mayo *et al*, 2001; Olivotto *et al*, 2001; Rayson *et al*, 2004). In the United Kingdom, however, the introduction of the 2-week rule for urgent referrals has generated much research into referral for presumptive cancer, with results indicating that the majority of patients urgently referred with symptoms suggestive of cancer are seen by a consultant within 14 days of referral (Debnath *et al*, 2002; Scott *et al*, 2006). Although studies also report time intervals following referral (Allgar and Neal, 2005; Neal *et al*, 2007), variations across studies in the precise definitions of the time intervals as well as the data capture methods make direct comparisons difficult. Standardising wait time terminology will permit improved comparison over time and across jurisdictions. We present our results here as medians as well as means and standard deviations to allow for the greatest flexibility in any future comparisons.

The time interval between the date of referral and confirmed diagnosis was much shorter for patients with presumptive lung cancer than for patients with presumptive colorectal or prostate cancer. The longer intervals for colorectal and prostate patients may reflect the subtle and non-specific nature of symptoms (Roncoroni *et al*, 1999; Allgar and Neal, 2005). Conversely, signs and symptoms leading to referral for investigation of possible lung cancer are strongly predictive and there is a readily available diagnostic test (e.g., chest X-ray). Indeed, lung patients in our study were most likely to have cancer, with the most common referral reason being suspicious lung nodule(s) or lesion or mass on diagnostic imaging. Nonetheless, differences in the presentation of signs/symptoms and diagnostic processes associated with different cancers underlines the value of the present data for informing policies and standards on diagnostic intervals, and supports the need for a more nuanced approach when setting wait time standards.

Patients with presumptive colorectal cancer show an interesting pattern. Those with cancer both saw the consultant and were informed of their diagnosis sooner than those without cancer (median 9 days *vs* 48 days; median 13 days *vs* 72 days, respectively), suggesting that clinicians have an effective process for triaging patients referred for diagnostic assessment. Similar effects have been reported for Canadian breast patients (Olivotto *et al*, 2001). Surprisingly, this triaging is not necessarily apparent in data gathered because the implementation of the United Kingdom's urgent 2-week rule, which was intended to facilitate the triage system. For example, though the majority of suspected colorectal cancer patients may see a consultant within the 2-week period (Debnath *et al*, 2002; Scott *et al*, 2006; Neal *et al*, 2007), those patients can wait considerably longer for their diagnosis once entering the hospital system (Neal *et al*, 2007).

The time intervals between referral and confirmed diagnosis for all patients were longer than the Canadian Strategy for Cancer Control's (CSCC) recommended 4-week maximum between the first visit to a physician and diagnosis (2002). In fact, colorectal and prostate patients waited longer than 10 weeks between referral for suspected cancer and being informed of their diagnosis. The Canadian Society for Surgical Oncology (n.d.) recommends that the time from referral to first consultation should not exceed 2 weeks; this recommendation is consistent with the United Kingdom 2-week rule for urgent suspected cancers (Department of Health, 2000a). Only lung patients in our study came close to meeting these recommendations.

The prolonged peri-diagnostic waits experienced by our colorectal and prostate patients, as well as the lengthy wait times reported by others (Spurgeon *et al*, 2000; Porter *et al*, 2005), point towards the need to further understand the nature and dynamics of cancer referrals and diagnoses. Canada, Australia, the United Kingdom, and many European countries employ a ‘gatekeeping’ model of health care (Gérvas *et al*, 1994; Brennan and Houssami, 2006), whereby primary care providers act as the point of entry each time care is sought for a health problem. The best way to use this model to serve patients requiring prompt specialist care is an issue requiring further investigation. The CSCC (2006), Australia's National Service Improvement Framework for Cancer (National Health Priority Action Council, 2006), and the United Kingdom's new Cancer Reform Strategy (Department of Health, 2007) all emphasise the importance of earlier diagnoses for improved cancer control, including the need to further study wait times in cancer diagnosis to strengthen the primary care provider's ability to facilitate early diagnosis.

We found no differences in HRQL and health status between patients with cancer and those without. This may reflect the unexplained signs and symptoms and continued uncertainty that those without cancer presumably still had, despite non-malignant findings. Others have found that subjective measures of care (eg, satisfaction) are more sensitive to changes in the interval from diagnosis to surgery than to changes in the peri-diagnostic period (Porter *et al*, 2005). Perhaps patients' HRQL and anxiety also escalate in the time between diagnosis and subsequent treatment events, whereas our assessment largely represents the peri-diagnostic period.

Although we measured wait time intervals in the peri-diagnostic period, we do not provide any information on patient factors contributing to these intervals. Another limitation of this study is that the impact of wait times on outcomes was not determined. This is recognised as an important missing element in our knowledge about wait times (CSCC, 2002). As our objective was to describe wait time intervals for patients with suspected cancer, we did not obtain additional clinical and pathological data on the tumours nor did we follow patients after treatment to measure outcomes. Finally, our study examined time intervals at one regional hospital in Ontario, which may not be representative of wait times throughout Canada or indeed other primary care-based health care systems.

Despite the heightened political interest in timely access to health care, acceptable and effective wait time benchmarks for the peri-diagnostic period have not been established. To our knowledge, this study is the first to measure prospectively diagnostic wait time intervals for suspected colorectal, lung, and prostate cancers in Canada. As such, it provides important descriptive data to assess and determine benchmark measures. Furthering the work of wait time initiatives (Wait Time Alliance, 2005) is vital to ensure common definitions are established to aid in the measurement and comparison of wait times, to manage wait times more effectively, and to monitor changes over time.

## Figures and Tables

**Table 1:** Participant characteristics (*n*=350)

	**Colorectal**			**Lung**			**Prostate**		
	**All^a^**	**Cancer^b^**	**No cancer^c^**	**All^a^**	**Cancer^b^**	**No cancer^c^**	**All^a^**	**Cancer^b^**	**No cancer^c^**
Number (%)	133	9 (6.8)	124 (93.2)	101	81 (80.2)	20 (19.8)	116	41 (35.3)	75 (64.7)
Age; mean (s.d.)	62.3 (11.2)	73.8 (8.8)	61.4 (11.0)	65.0 (11.8)	66.4 (11.6)	59.5 (11.1)	65.5 (9.4)	65.6 (9.8)	65.5 (9.2)
									
*Sex (no.)*									
Male	71	7	64	58	48	10	116	41	75
Female	62	2	60	43	33	10	—	—	—
									
*Marital status (no.)*									
Single	8	0	8	6	4	2	8	1	7
Married	103	7	96	63	52	11	91	34	57
Divorced	13	0	13	13	10	3	14	5	9
Widowed	9	2	7	19	15	4	3	1	2
									
*Comorbidity (no.)* d									
0	101	4	97	55	41	14	84	31	53
1	22	3	19	22	21	1	23	5	18
2	7	1	6	11	8	3	6	4	2
>3	3	1	2	13	11	2	3	1	2
									
*Referring physician (no.)*									
Family physician	116	9	107	74	63	11	103	38	65
Surgeon	4	0	4	3	2	1	1	0	1
Specialist^e^	1	0	1	5	2	3	4	2	2
Other	12	0	12	19	14	5	8	1	7

a All patients referred with symptoms suggestive of cancer.

b Patients for whom cancer was confirmed.

c Patients for whom cancer was confuted.

d Charlson comorbidity scores: range from 0 to 10 with a higher score indicating greater number of comorbid conditions (Charlson *et al*, 1987).

e Specialists included gastroenterologists, respirologists, and urologists.

**Table 2:** Time intervals to diagnosis, surgery, and oncology consultation (*n*=350)

	**Colorectal**			**Lung**			**Prostate**		
**Time interval (days)**	**All^a^ (*n*=133)**	**Cancer^b^ (*n*=9)**	**No cancer^c^ (*n*=124)**	**All^a^ (*n*=101)**	**Cancer^b^ (*n*=81)**	**No cancer^c^ (*n*=20)**	**All^a^ (*n*=116)**	**Cancer^b^ (*n*=41)**	**No cancer^c^ (*n*=75)**
*1. Referral to patient informed of diagnosis*									
Mean (s.d.)	81.3 (67.7)	33.8 (48.9)	84.8 (67.7)	45.2 (32.4)	43.5 (31.7)	52.2 (35.1)	82.3 (42.9)	91.3 (37.4)	77.5 (45.1)
Median (IQR)	71 (30–110)	13 (8–35)	72 (32–111)	37 (29–49)	35 (28–49)	42 (36–49)	81 (56–100)	85 (73–100)	77 (40–100)
									
*2. Referral to confirmed diagnosis* d									
Mean (s.d.)	75.2 (63.6)	31.0 (45.2)	78.4 (63.7)	35.5 (31.5)	33.6 (31.1)	43.1 (32.6)	68.0 (38.3)	72.6 (35.0)	65.4 (40.0)
Median (IQR)	66 (29–103)	13 (8–32)	71 (30–104)	28 (21–37)	28 (19–35)	33 (26–45)	65.5 (44–84)	66 (58–80)	64 (37–84)
									
*3. Referral to first diagnostic consult* e									
Mean (s.d.)	55.1 (48.4)	13.4 (11.6)	58.2 (48.7)	13.9 (11.4)	12.6 (9.9)	19.2 (15.1)	38.0 (24.6)	40.4 (28.0)	36.7 (22.6)
Median (IQR)	43 (17–79)	9 (7–13)	48 (20–81)	12 (6–19)	11 (6–18)	14 (10–26)	35 (25–49)	35 (29–50)	34 (24–49)
									
*4. First diagnostic consult to patient informed of diagnosis*									
Mean (s.d.)	26.6 (42.3)	20.3 (51.9)	27.0 (41.7)	31.4 (32.3)	31.0 (32.6)	33.1 (31.7)	44.3 (31.7)	50.9 (22.1)	40.8 (35.5)
Median (IQR)	0 (0–40)	0 (0–11)	1.5 (0–44)	25 (14–35)	25 (13–37)	24 (19–35)	45 (28–58)	47 (37–58)	39 (0–58)
									
*5. Confirmed diagnosis to surgery/decision for no surgery*		n=9			n=73			n=41	
Mean (s.d.)	—	33.9 (23.3)	—	—	22.7 (18.8)	—	—	61.6 (45.7)	—
Median (IQR)	—	23 (17–43)	—	—	16 (10–28)	—	—	54 (19–96)	—
									
*6. Referral to surgery/decision for no surgery*		n=9			n=77			n=41	
Mean (s.d.)	—	64.9 (42.0)	—	—	54.9 (39.2)	—	—	134.2 (62.2)	—
Median (IQR)	—	64 (30–76)	—	—	45 (35–63)	—	—	128 (80–168)	—
									
*7. Referral to surgery* f		n=8			n=23			n=20	
Mean (s.d.)	—	53.1 (24.3)	—	—	72.9 (51.3)	—	—	170.2 (59.9)	—
Median (IQR)	—	54.5 (30–76)	—	—	57 (44–91)	—	—	162 (120–215)	—
									
*8. Surgery to oncology consult/ decision for no consult*		n=3			n=20			n=6	
Mean (s.d.)	—	21.0 (18.2)	—	—	39.7 (33.9)	—	—	18.7 (21.8)	—
Median (IQR)	—	11 (10–42)	—	—	35 (22–47)	—	—	10.5 (8–12)	—
									
*9. Referral to initiation of first treatment* g		n=9			n=81			n=41	
Mean (s.d.)	—	64.0 (41.8)	—	—	55.5 (38.9)	—	—	113.6 (52.4)	—
Median (IQR)	—	64 (30–75)	—	—	45 (35–64)	—	—	94 (79–135)	—

a All patients referred with symptoms suggestive of cancer.

b Patients for whom cancer was confirmed.

c Patients for whom cancer was confuted.

d Date of pathology, radiology, or procedural report confirming cancer diagnosis.

e Date of first diagnostic consult was defined as the date first seen by a consultant or the date of the first relevant investigation initiated by consultant, whichever came first.

f Includes only those patients who underwent surgery.

g First treatment was defined as neoadjuvant chemotherapy, surgery if no preoperative treatment was required, chemotherapy, radiotherapy, or a decision for no treatment.

**Table 3:** Time interval from date of referral to date patient informed of diagnosis: comparison of patients with and without cancer

	**Cancer^a^**	**No cancer^b^**			
	**days; mean (s.d.)**		**Difference (95% CI)**	***t*-test *P*-value**	**Wilcoxon *P*-value**
Colorectal (*n*=133)	33.8 (48.9)	84.8 (67.7)	51 (5.4–96.6)	0.03	0.003
Lung (*n*=101)	43.5 (31.7)	52.2 (35.1)	8.7 (−7.3–24.7)	0.28	0.14
Prostate (*n*=116)	91.3 (37.4)	77.5 (45.0)	−13.8 (−30.2–2.6)	0.10	0.09
All (*n*=350)	57.8 (41.4)	79.3 (58.9)	21.5 (10.0–33.0)	0.0001	0.0007

a Patients for whom cancer was confirmed.

b Patients for whom cancer was confuted.

**Table 4:** Comparison of health status and quality of life 1 week after diagnosis for patients with and without cancer

**Parameter; mean (s.d.)**	**All^a^**	**Cancer^b^**	**No cancer^c^**	**Difference (95% CI)**	***P*-value**
*SF36 PCS*					
Colorectal	46.29 (10.66)	41.54 (7.52)	46.58 (10.78)	5.04 (−3.84–13.92)	0.26
Lung	37.81 (11.75)	36.94 (11.64)	41.37 (11.88)	4.43 (−2.06–10.93)	0.18
Prostate	50.27 (10.01)	52.04 (11.04)	49.23 (9.28)	−2.81 (−6.80–1.18)	0.16
*P*-value (difference between disease sites)	ANOVA=0.0001 kw=0.0001				
					
*SF36 MCS*					
Colorectal	48.75 (10.40)	49.28 (8.79)	48.71 (10.53)	−0.57 (−9.29–8.15)	0.90
Lung	42.71 (15.14)	42.54 (14.34)	43.40 (18.59)	0.86 (−7.61–9.32)	0.84
Prostate	52.57 (9.08)	50.49 (10.11)	53.80 (8.25)	3.31 (−0.29–6.90)	0.07
*P*-value (difference between disease sites)	ANOVA=0.0001 kw=0.0001				
					
*HADS Anxiety*					
Colorectal	3.37 (1.09)	3.17 (0.98)	3.38 (1.09)	0.22 (−0.69–1.13)	0.63
Lung	3.63 (1.18)	3.63 (1.18)	3.63 (1.20)	0.00 (−0.66–0.65)	0.99
Prostate	2.87 (0.88)	2.87 (0.92)	2.87 (0.87)	0.02 (−0.33–0.38)	0.90
*P*-value (difference between disease sites)	ANOVA=0.0001 kw=0.0001				
					
*HADS Depression*					
Colorectal	5.70 (2.1)	4.83 (0.98)	5.75 (2.15)	0.92 (−0.83–2.68)	0.30
Lung	6.93 (2.84)	7.13 (2.84)	6.12 (2.75)	−1.01 (−2.58–0.56)	0.20
Prostate	5.06 (1.57)	4.97 (1.77)	5.10 (1.46)	0.16 (−0.48–0.79)	0.62
*P*-value (difference between disease sites)	ANOVA=0.0822 kw=0.0825				
					
*EORTC QLQ C-30*					
*Physical functioning*					
Colorectal	85.52 (18.55)	73.33 (19.78)	86.26 (18.31)	12.93 (−2.40–28.26)	0.10
Lung	65.27 (27.34)	63.79 (27.59)	71.25 (26.30)	7.46 (−7.74–22.65)	0.33
Prostate	91.74 (14.96)	91.97 (17.52)	91.34 (13.68)	−0.63 (−6.69–5.45)	0.84
*P*-value (difference between disease sites)	ANOVA=0.0001 kw=0.0001				
					
*Role functioning*					
Colorectal	82.06 (23.54)	72.22 (27.22)	82.66 (23.32)	10.44 (−9.18–30.05)	0.29
Lung	53.09 (39.89)	53.59 (39.80)	51.04 (41.49)	−2.55 (−24.84–19.74)	0.82
Prostate	90.98 (19.45)	88.46 (22.99)	92.04 (17.49)	3.58 (−4.28–11.44)	0.37
*P*-value (difference between disease sites)	ANOVA=0.0001 kw=0.0001				
					
*Emotional functioning*					
Colorectal	77.78 (19.70)	76.39 (12.27)	77.86 (20.10)	1.47 (−15.03–17.98)	0.86
Lung	67.59 (26.48)	67.82 (25.43)	66.67 (31.33)	−1.15 (−15.96–13.65)	0.88
Prostate	82.72 (18.38)	79.27 (18.82)	84.20 (18.24)	4.93 (−2.44–12.30)	0.19
*P*-value (difference between disease sites)	ANOVA=0.0001 kw=0.0001				
					
*Cognitive functioning*					
Colorectal	85.71 (17.52)	91.67 (13.94)	85.35 (17.70)	−6.31 (−20.94–8.31)	0.39
Lung	72.84 (25.34)	71.79 (26.34)	77.08 (20.97)	5.29 (−8.83–19.40)	0.46
Prostate	88.07 (16.99)	85.47 (19.93)	89.30 (15.27)	3.83 (−3.01–10.67)	0.27
*P*-value (difference between disease sites)	ANOVA=0.0001 kw=0.0001				
					
*Social functioning*					
Colorectal	83.97 (22.51)	75.00 (22.97)	84.52 (22.58)	9.52 (−9.25–28.30)	0.32
Lung	63.37 (34.00)	63.33 (34.38)	63.54 (33.45)	0.21 (−18.79–19.21)	0.98
Prostate	91.74 (14.81)	89.32 (17.31)	92.79 (13.37)	3.47 (−2.49–9.43)	0.25
*P*-value (difference between disease sites)	ANOVA=0.0001 kw=0.0001				
					
*Global Health Status*					
Colorectal	69.47 (18.64)	59.72 (17.81)	70.07 (18.61)	10.35 (−5.15–25.84)	0.19
Lung	51.34 (25.53)	50.51 (25.25)	54.69 (27.21)	4.18 (−10.07–18.42)	0.56
Prostate	76.99 (16.59)	75.00 (20.23)	77.61 (14.23)	2.61 (−4.05–9.27)	0.48
*P*-value (difference between disease sites)	ANOVA=0.0001 kw=0.0001				

PCS=physical component summary of Short-Form 36; kw=Kruskal–Wallis rank sum test; MCS=mental component summary of Short-Form 36; ANOVA=analyses of variance; EORTC QLQ C-30=European Organization of Research and Treatment of Cancer quality of life core questionnaire; HADS=hospital anxiety and depression scale.

a All patients referred with symptoms suggestive of cancer.

b Patients for whom cancer was confirmed.

c Patients for whom cancer was confuted.
